# Distribution of diabetes, hypertension and non-communicable disease risk factors among adults in rural Bangladesh: a cross-sectional survey

**DOI:** 10.1136/bmjgh-2018-000787

**Published:** 2018-11-12

**Authors:** Edward Fottrell, Naveed Ahmed, Sanjit Kumer Shaha, Hannah Jennings, Abdul Kuddus, Joanna Morrison, Kohenour Akter, Badrun Nahar, Tasmin Nahar, Hassan Haghparast-Bidgoli, A K Azad Khan, Anthony Costello, Kishwar Azad

**Affiliations:** 1 Institute for Global Health, University College London, London, UK; 2 Diabetic Association of Bangladesh, Dhaka, Bangladesh; 3 Maternal, Newborn, Child and Adolescent Health, World Health Organisation, Geneva, Switzerland

**Keywords:** epidemiology, diabetes, hypertension, community-based survey, cross-sectional survey, non-communicable disease, Bangladesh, rural

## Abstract

**Background:**

Non-communicable diseases (NCDs) are increasing in low-income settings. We conducted a survey of risk factors, blood pressure and blood glucose in rural Bangladesh and assessed variations by age, sex and wealth.

**Methods:**

We surveyed a random sample of 12 280 adults aged >30 years in 96 villages in rural Bangladesh. Fieldworkers measured blood glucose and conducted an glucose tolerance test with a repeat blood test 120 min post glucose ingestion. Blood pressure, anthropometric, socioeconomic, lifestyle and behavioural risk factors data were also collected. Data were analysed to describe the prevalence of diabetes, intermediate hyperglycaemia, hypertension and NCD risk factors by age, sex and wealth.

**Results:**

Women had higher levels of overweight or obesity and lower levels of physical activity and fruit and vegetable consumption than men; 63% of men used tobacco compared with 41.3% of women. Overweight or obesity and abdominal obesity (waist to hip ratio) increased with socioeconomic status (least poor vs most poor: OR (95% CI) 3.21 (2.51 to 4.11) for men and 2.83 (2.28 to 3.52) for women). Tobacco use, passive smoke exposure and salt consumption fell with increasing socioeconomic status in both sexes. Clustering of risk factors showed more than 70% of men and women reported at least three risk factors. Women in the least poor group were 33% more likely to have three or more risk factors compared with women in the most poor group (1.33 (95% CI 1.17 to 1.58)). The combined prevalence of impaired fasting glucose, impaired glucose tolerance and diabetes was 26.1% among men and 34.9% among women, and increased with age. The prevalence of prehypertension and hypertension was 30.7% and 15.9% among men and 27.2% and 22.5% among women, with similar rising prevalence with age.

**Conclusion:**

NCD risk factors, hyperglycaemia and raised blood pressure are an immediate health threat in rural Bangladesh. Initiatives to improve detection, treatment and prevention strategies are needed.

**Key questions**

**What is already known?**

- Low-income and middle-income countries suffer the largest burden of morbidity and mortality due to non-communicable diseases (NCDs).
- Description of risk factors, blood pressure and blood glucose in rural Bangladesh by age, sex and wealth is needed to better plan and evaluate intervention initiatives.

**What are the new findings?**

- Three in four adults in rural Bangladesh report three or more NCD risk factors.
- One in three adults in rural Bangladesh have intermediate hyperglycaemia or diabetes, and one in two adults have prehypertension or hypertension.

**What do the new findings imply?**

- The clear urgent need for prevention and treatment provides impetus for gender-sensitive, rural, community-based implementation of NCD policy and intervention programmes.

## Introduction

Approximately 80% of premature non-communicable disease (NCD) deaths occur in low-income and middle-income countries.^1^ This burden is driven by early life exposures^2–6^ and pandemics of overweight and obesity, tobacco use, air pollution, low levels of physical activity and high levels of salt intake, which disproportionately affect populations living in the world’s poorest countries.^1^ To address these burdens within resource-poor health systems, weak health information systems, highly unregulated private health sectors and ageing populations, it is first necessary to accurately measure and describe the burden of risk factors and disease.

The prevalence of NCD risk factors is thought to be high in Bangladeshi adults, with more than one-third of the population aged 25 years or older reporting three or more common risk factors.^7^ The 2010 Bangladesh NCD Risk Factor Survey found high levels of NCD risk factors and reported that approximately 4% of adults aged 25 years or older had diabetes mellitus, although this was based only on self-reported diagnosis by a health professional.^8^ Among those aged over 35, the Bangladesh Demographic and Health Survey (BDHS) reported that 24% had abnormal fasting glucose and 8% had diabetes according to fasting blood glucose results.^9^ Analysing the data from the BDHS, Akter *et al* 10 found higher rates of diabetes according to age but no significant difference in male and female rates of diabetes or impaired fasting glucose (IFG) levels. While there was a significant difference between the poorest and richest households in diabetic status rates (6.4% of the poorest households and 19.2% richest households), the difference was considerably lower for IFG levels (19.7% and 23.5%, respectively). Other studies report varying levels of hyperglycaemia (including IFG, impaired glucose tolerance (IGT) and/or diabetes), ranging from 9% to 30% depending on study setting, level of urbanisation, age of study population, methods of blood glucose assessment and timing of study.^11–18^ Furthermore, a recent review by Rahman *et al* 19 describes a threefold to fourfold increase in diabetes prevalence between 1992 and 2015, with levels expected to reach 23.6% in men and 33.5% in women by 2030 That is three to four times greater than the 2016 age-standardised global prevalence estimate of 8.5% for adults.^20^

The Bangladesh NCD Risk Factor Survey also found that one-third of adults self-reported a medical diagnosis of hypertension,^8^ although study estimates of hypertension prevalence range from 11% to 40%, with increases generally corresponding with more urban locations, older study populations and increasing socioeconomic status, recency of survey and individual comorbidities such as diabetes.^7 21–27^ Analyses of the 2011 BDHS data report the prevalence of hypertension to be higher among women than men.^28^ Rahman *et al* 19 describe an increase in the prevalence of hypertension among men and women of approximately 50% and 85%, respectively, between 1992 and 2015.

Socioeconomic inequalities in hyperglycaemia, raised blood pressure and NCD risk factors have been described, with a high burden of NCDs observed among low-wealth quintile populations in rural areas and wealthier populations in urban areas.^29^ Further, evidence suggests that increasing levels of wealth and educational attainment are associated with increased likelihood of having hypertension and hyperglycaemia,^26 28 30^ and individuals with comorbid diabetes and hypertension from low socioeconomic groups are less likely to be diagnosed, receive treatment or control their blood pressure.^25^

This paper presents an epidemiological survey conducted by the Diabetic Association of Bangladesh and University College London. As part of a cluster randomised controlled trial in a rural Bangladeshi population,^31^ we conducted a large, community-based, cross-sectional survey of NCD risk factors, blood glucose and blood pressure to describe the prevalence and variance by age, sex and socioeconomic status, and to discuss awareness, treatment and control of diabetes and hypertension in this setting.

## Methods

The study included 96 villages in four rural upazilas—Nagarkanda, Boalmari, Saltha and Madhukhali—in Faridpur District, located south of Dhaka. Faridpur District has a population of over 1.7 million people in an area of just over 2000 square kilometres and is situated on the banks of Padma River. The district has a mainly agricultural-based economy, with the main crops being jute and rice. As in the rest of Bangladesh, primary care is provided at Union Health and Family Welfare Centres and at community clinics. Inpatient and outpatient services are provided at subdistrict (upazila) health complexes and hospitals, and tertiary care is provided at district hospitals and medical college hospitals.^32^ Private service providers are also present in the area, including a Diabetic Association of Bangladesh Medical College in Faridpur town. Nevertheless, access to facilities and trained healthcare providers, short supplies of medicines and low responsiveness of services remain as challenges in Faridpur District.

The study population includes male and non-pregnant female permanent residents of 96 villages aged 30 years or more. A person is considered a permanent resident of a village if they normally live in that village. A sampling frame of all eligible participants was developed between October and December 2015 by listing all households and their eligible members within each of the study villages. A sample of 143 adults aged 30 years and above was selected from this sampling frame using multistage random sampling. In the first stage, 143 households with at least one eligible adult resident were selected using probability proportional to size sampling. In the next stage, a single eligible adult was selected from each of the 143 households for inclusion in the survey using simple random sampling. The sample size was determined by trial requirements described elsewhere.^31^ In terms of epidemiological analysis, the total sample size allows estimation of the true population prevalence of intermediate hyperglycaemia and diabetes mellitus with 99% confidence and an accuracy of between 1% and 2%.

Data were collected by 16 teams of fieldworkers that comprised one male and one female with at least secondary education who were recruited locally and selected through a written assessment and interview. All fieldworkers underwent 12 days of physician-led training on the survey methods and how to take physical measurements, followed by 2 weeks of supervised field practice and daily debriefs in villages in Faridpur that were not included in the study. This was followed by 3 days of refresher training before the start of the survey on 23 January 2016. Data collectors were supervised by four field supervisors with experience in survey methods. Each supervisor was responsible for four data collection teams, spending half a day observing and verifying data within each team at least every 2 days. Within each village, teams were aided by a village assistant, usually a young man, who received a daily payment to coordinate study participants and assist data collectors in their duties.

All sampled individuals in a single cluster were informed of the anthropometric, blood glucose and blood pressure measurement requirements of the study and, following consent, were requested to attend a local centre on the morning of a specified day following an overnight fast. Centres were established by the field team for the purposes of the study and were at a central, convenient location in the village. Physical measurements were recorded by data collection teams at the testing centres. Blood glucose was measured using the OneTouch Ultra Glucometer (LifeScan, Milpitas, California) in whole blood obtained by finger prick from capillaries in the middle or ring finger after an overnight fast. All individuals then received a 75 g glucose load dissolved in 250 mL of water. A 2-hour postprandial repeat capillary blood test was then conducted to determine glucose tolerance status and differentiate between individuals with intermediate hyperglycaemia and those with diabetes according to the WHO criteria (table 1).

**Table 1:** Glycaemic definitions and diagnostic criteria

Definition	Diagnostic criteria
Normoglycaemia	Fasting plasma glucose <6.1 mmol/L
Intermediate hyperglycaemia	
Impaired fasting glucose	Fasting plasma glucose ≥6.1 mmol/L to <7.0 mmol/L AND 2-hour postingestion of 75 g glucose load plasma glucose <7.8 mmol/L.
Impaired glucose tolerance	Fasting plasma glucose<7.0 mmol/L AND 2-hour postingestion of 75 g glucose load plasma glucose ≥7.8 mmol/L to <11.1 mmol/L.
Type 2 diabetes mellitus	Fasting plasma glucose ≥7.0 mmol/L OR* 2-hour postingestion of 75 g glucose load plasma glucose >11.1 mmol/L.

Adapted from WHO 2006^59^.

*Diabetes cannot be excluded without 2-hour post oral glucose load test.

Sitting blood pressure was measured using the Omron HBP-1100 Professional Blood Pressure Monitor (Kyoto, Japan). Two measurements were taken at approximately 5 min intervals and the respondent’s blood pressure obtained by taking the average of these measurements. Measurements of height, weight, and waist and hip girth were taken with light clothes and without shoes. The weighing tools were calibrated daily by known weight. For height, the subject stood in erect posture vertically touching the occiput, back, hip and heels on a wall while gazing horizontally in front and keeping the tragus and lateral orbital margin in the same horizontal plane. Waist girth was measured by placing a plastic tape horizontally mid-way between the 12th rib and the iliac crest on the mid-axillary line. Similarly, hip circumference was measured by taking the extreme end posteriorly and the symphysis pubis anteriorly. Physical measures were recorded onto specifically designed paper forms and later entered into Samsung Galaxy Grand Prime large screen smartphones using ODK Collect with logic and range-check controls.^33^

We collected detailed information on the sociodemographic characteristics of all consenting individuals using a structured survey instrument adapted from the 2010 Bengali WHO Stepwise tool^8 34^ and the 2011 BDHS,^9^ which was designed to measure the background demographic and socioeconomic characteristics, lifestyle and behavioural risk factors among study participants. Questionnaire data were gathered using an ODK form on the mobile phone and linked to the physical measurement data using unique personal identification numbers. Collection of questionnaire data took place at the respondent’s home before or after the physical measurements or at the time of physical measurement in the testing centre. Data quality control measures were implemented within the ODK system (eg, range and consistency checks) and through repeat measures by supervisors on a random basis and where outlier data were recorded. Data were uploaded from mobile phones to the supervisors’ laptop every 2 days and then transferred to a central database at the Faridpur field office for further data checks and quality control before being transferred on a weekly basis to the main project office in Dhaka, where final data checking was implemented, with any data queries being referred back to the field for verification and remeasurement if necessary.

Analyses were restricted to those who provide at least partial data in both the physical measurement survey and the interview survey. Descriptive analysis summarised study population characteristics (sex, 10-year age categories, marital status, education, literacy, occupation, household wealth and religion), the prevalence of disease indicators (IFG, IGT, diabetes, prehypertension and hypertension), and risk factors for overweight and obesity (body mass index (BMI) ≥23 using cut-off values for South Asian populations),^35^ abdominal obesity (waist to hip ratio >0.85 for women and ≥0.9 for men),^36^ tobacco use, exposure to passive tobacco smoke, inadequate fruit and vegetable intake (defined as less than five servings per day), salt intake (defined as additional salt added to every meal after it has been cooked), and inadequate physical activity (defined as less than 150 min of physical activity per week). Clustering of risk factors was defined as three or more risk factors reported in the same individual, as has been used in previous studies.^7^

Households were categorised into five socioeconomic quintiles using a wealth index derived from principal components analysis of the household’s ownership of assets, housing characteristics and sanitation facilities. Hyperglycaemia (IFG, IGT and diabetes) was based on blood glucose concentration categorised according to the WHO diagnostic criteria (table 1) or self-reported diagnosis of diabetes by a medical professional. Prehypertension was defined as systolic blood pressure ≥120 mm Hg and <140 mm Hg or diastolic blood pressure ≥80 mm Hg and <90 mm Hg. Hypertension was defined as systolic blood pressure ≥140 mm Hg or diastolic blood pressure ≥90 mm Hg, or self-reported diagnosis of hypertension by a medical professional or self-reported current treatment with antihypertensive medication. Risk factor and disease outcomes were presented by age and wealth quintile and stratified by sex. Associations between age and wealth quintile and risk factors and disease were assessed using logistic regression adjusted for potential confounding factors of education, occupation, literacy, marital status, and either wealth or age group, respectively. Awareness of diabetic and hypertensive status was determined by the proportion of individuals meeting the diagnostic criteria who reported a prior knowledge of their disease status. Treatment for diabetes and hypertension was based on ever receiving treatment or advice for diabetes or hypertension from a medical professional among those who had a previous medical diagnosis of either disease. Control was defined as individuals with diabetes or hypertension who had prior knowledge of their disease status and did not meet the objective diagnostic criteria in our survey measures of blood glucose or blood pressure. All analyses were carried out in Stata V.13 and adjusted for the clustered and stratified survey design and weighted to account for the unequal probability of selection of a fixed number of individuals within villages of unequal size using the ‘svy’ command in Stata.

Participation in the surveys was voluntary and informed consent was obtained from all participants before any data were collected. No reimbursements or incentives were given to participants. Individuals identified with raised blood glucose or raised blood pressure indicating diabetes or hypertension, respectively, were advised to visit qualified health professionals for further testing and care advice.

## Results

### Response rate and study population

Using the sampling frame we developed a target sample list of 13 684 individuals. Note that this number is slightly lower than the expected target of 143 individuals in each of the 96 villages (13 728 individuals) as 2 villages only had 128 and 114 eligible individuals living in separate households, respectively.

Data were collected from a total of 12 280 individuals (5669, 46.2% male; 6471, 52.7% female) out of a target of 13 684 between January and March 2016, representing an overall response rate of 89.5%. A total of 12 047 (87.8%) individuals (5615, 46.6% male; 6432, 53.4% female) provided at least partial data in the physical measurement survey and the interview survey and are included in the following analysis. Using sampling frame data, it was possible to explore age bias in response rates. Male non-responders were younger (mean age 45.4 years (SD 13.0)) than male responders (48.9 years (SD 13.8)); p<0.001). Conversely, female non-responders were slightly older (49.3 (SD 17.8)) compared with female responders (46.6 (SD 13.6); p<0.001).

Study population characteristics are presented in table 2 and show that approximately 90% of the population were Muslim. Overall, the population had low levels of education and literacy, although there were notable differences between men and women and by age group. Two-thirds of men worked in manual occupations, whereas 94% of women had no paid employment. The proportion of women who were married at the time of the survey decreased considerably with increasing age.

**Table 2:** Study population characteristics

		Men						Women					
		Age (years)					Total	Age (years)					Total
		30–39	40–49	50–59	60–69	70+		30–39	40–49	50–59	60–69	70+	
Education													
No formal education	n (%)	516 (29.3)	584 (41.9)	584 (51.7)	256 (54.6)	213 (51.6)	2460 (43.1)	678 (28.5)	970 (57.9)	823 (71.1)	739 (83.8)	337 (92.8)	3547 (55.0)
Incomplete primary	n (%)	511 (30.7)	332 (25.3)	244 (22.2)	212 (20.5)	81 (21.9)	1380 (25.1)	655 (27.5)	397 (24.7)	213 (18.7)	95 (11.4)	17 (4.9)	1377 (21.7)
Complete primary or above	n (%)	689 (40.0)	445 (32.8)	296 (26.1)	256 (24.9)	105 (26.4)	1791 (31.7)	1029 (43.9)	296 (17.5)	117 (10.3)	40 (4.8)	10 (2.3)	1492 (23.3)
Literate	n (%)	957 (56.2)	957 (56.1)	605 (45.0)	402 (35.5)	360 (34.8)	2465 (43.9)	1388 (59.2)	1388 (59.2)	467 (28.2)	213 (18.4)	78 (9.2)	2164 (33.9)
Currently married	n (%)	1671 (97.3)	1352 (99.2)	1111 (98.8)	994 (96.4)	345 (87.4)	5473 (97.2)	2284 (96.7)	1486 (89.9)	842 (73.9)	399 (45.7)	67 (18.5)	5078 (79.4)
Occupation													
Unemployed	n (%)	13 (0.7)	19 (1.5)	75 (6.9)	255 (24.8)	221 (57.2)	583 (10.6)	2242 (94.6)	1568 (94.4)	1079 (93.7)	835 (95.5)	355 (97.9)	6079 (94.7)
Manual	n (%)	1278 (74.4)	985 (72.4)	792 (70.5)	596 (58.0)	140 (34.0)	3791 (67.3)	55 (2.5)	57 (3.3)	52 (4.4)	29 (3.2)	5 (1.1)	198 (3.1)
Professional	n (%)	425 (24.8)	357 (26.1)	257 (22.7)	180 (17.2)	37 (8.7)	1256 (22.1)	65 (3.0)	38 (2.3)	22 (1.8)	10 (1.3)	3 (1.0)	138 (2.3)
Wealth quintiles													
Most poor	n (%)	363 (20.9)	225 (16.3)	181 (16.2)	175 (17.2)	72 (17.6)	1016 (17.9)	503 (20.7)	288 (17.0)	286 (25.0)	231 (25.7)	86 (23.1)	1394 (21.3)
2	n (%)	329 (19.5)	266 (20.0)	215 (18.9)	198 (19.2)	62 (15.5)	1070 (19.1)	539 (23.1)	330 (19.7)	223 (19.5)	191 (23.0)	72 (20.7)	1355 (21.4)
3	n (%)	350 (20.1)	305 (22.0)	259 (23.3)	212 (20.6)	73 (18.0)	1199 (21.2)	442 (19.1)	364 (21.9)	190 (16.7)	165 (18.7)	63 (17.4)	1224 (19.2)
4	n (%)	362 (21.0)	305 (22.7)	258 (22.8)	210 (19.7)	70 (17.6)	1205 (21.3)	429 (17.8)	329 (20.0)	227 (19.4)	132 (14.8)	65 (16.5)	1182 (18.2)
Least poor	n (%)	312 (18.5)	260 (19.0)	211 (18.7)	236 (23.4)	122 (31.4)	1141 (20.5)	449 (19.3)	352 (21.5)	227 (19.5)	155 (17.7)	78 (22.3)	1261 (19.9)
Muslim	n (%)	1572 (91.5)	1571 (91.4)	1224 (90.0)	994 (89.3)	916 (88.6)	363 (91.5)	2168 (92.1)	2169 (92.1)	1507 (91.0)	1039 (90.5)	797 (91.5)	332 (91.3)
Total	n (%)	1716 (30.0)	1361 (13.6)	1124 (20.3)	1031 (18.4)	399 (7.1)	5631 (46.9)	2362 (36.8)	1663 (26.0)	1153 (17.9)	874 (13.8)	364 (5.6)	6416 (53.1)

### Risk factors

**Figure 1:**
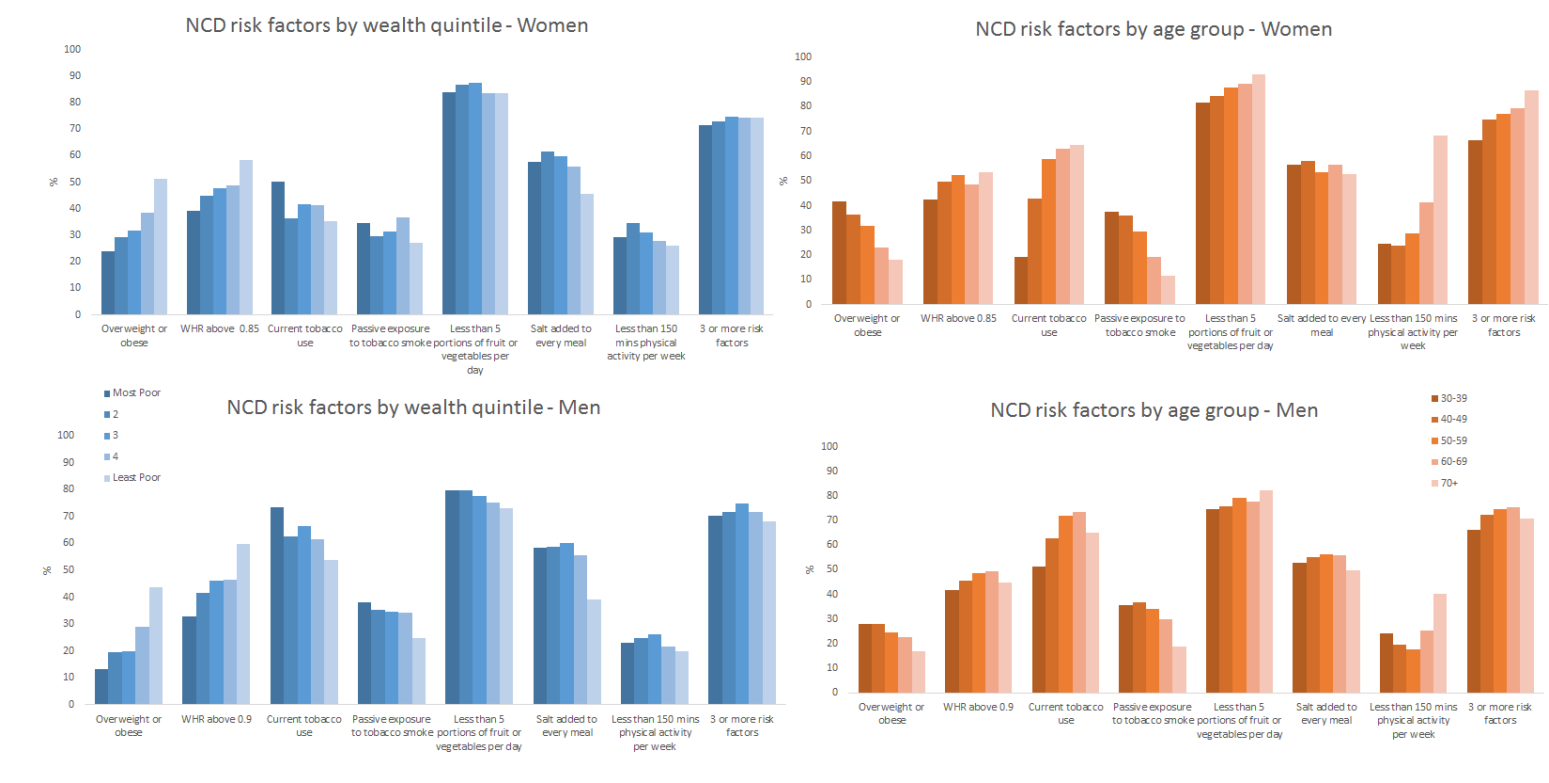
NCD risk factor prevalence by wealth quintile, age group and sex among adults aged 30 years and older in rural Faridpur. NCD, non-communicabledisease; WHR, waist to hip ratio.

Table 3 and figure 1 present the level of NCD risk factors and risk factor clustering by wealth quintile and sex. Overall, compared with men, women had higher levels of overweight or obesity and lower levels of physical activity and fruit and vegetable consumption. Men in all wealth groups had higher levels of tobacco use, with 63.3% of all men currently using tobacco compared with 41.3% of women. Method of tobacco use differed substantially between sexes, with 87% of men smoking tobacco compared with just 1% of women, who generally consumed tobacco orally or nasally.

**Table 3:** Prevalence and ORs of non-communicable disease risk factors and risk factor clustering by sex and wealth quintile among adults aged 30 years and older in rural Faridpur

	Overweight or obese		Abdominal obesity		Current tobacco use		Passive exposure to tobacco smoke		<5 daily portions of fruits or vegetables		Salt added to every meal		<150 min physical activity per week		>3 risk factors	
	%	OR (95% CI)	%	OR (95% CI)	%	OR (95% CI)	%	OR (95% CI)	%	OR (95% CI)	%	OR (95% CI)	%	OR (95% CI)	%	OR (95% CI)
**Men**																
Wealth quintile*																
Most poor	13.1	1	32.7	1	73.3	1	38.2	1	79.7	1	58.3	1	23	1	70.2	1
2	19.6	1.51 (1.18 to 1.92)	41.6	1.40 (1.14 to 1.71)	62.6	0.59 (0.47 to 0.75)	35.3	0.88 (0.72 to 1.09)	79.8	1.02 (0.81 to 1.29)	58.9	1.05 (0.84 to 1.32)	24.7	1.12 (0.87 to 1.45)	71.7	1.06 (0.85 to 1.32)
3	19.9	1.39 (1.10 to 1.75)	46.2	1.58 (1.29 to 1.93)	66.5	0.73 (0.61 to 0.87)	34.7	0.89 (0.72 to 1.11)	77.7	0.93 (0.72 to 1.20)	60	1.15 (0.91 to 1.45)	26.3	1.21 (0.91 to 1.62)	74.7	1.23 (0.97 to 1.55)
4	29.1	2.10 (1.69 to 2.61)	46.4	1.49 (1.21 to 1.84)	61.5	0.63 (0.51 to 0.77)	34.3	0.91 (0.71 to 1.18)	75	0.83 (0.61 to 1.14)	55.6	1.00 (0.76 to 1.32)	21.5	0.88 (0.62 to 1.23)	71.8	1.06 (0.85 to 1.31)
Least poor	43.8	3.21 (2.51 to 4.11)	59.7	2.13 (1.67 to 2.70)	53.9	0.51 (0.42 to 0.63)	24.8	0.66 (0.50 to 0.87)	72.9	0.81 (0.58 to 1.14)	39.3	0.58 (0.45 to 0.75)	19.9	0.66 (0.49 to 0.89)	68.2	0.87 (0.68 to 1.11)
Age group†																
30–39	28	1	41.8	1	51.4	1	35.7	1	74.5	1	52.7	1	24.1	1	66.2	1
40–49	27.9	1.01 (0.85 to 1.21)	45.7	1.21 (1.03 to 1.42)	62.6	1.61 (1.39 to 1.87)	36.7	1.03 (0.87 to 1.22)	75.9	1.07 (0.90 to 1.26)	55	1.05 (0.90 to 1.23)	19.7	0.76 (0.62 to 0.93)	72.3	1.31 (1.12 to 1.54)
50–59	24.6	0.90 (0.73 to 1.11)	48.5	1.40 (1.19 to 1.65)	72	2.52 (2.12 to 2.99)	34.1	0.93 (0.78 to 1.10)	79.1	1.27 (1.04 to 1.54)	56.1	1.08 (0.92 to 1.27)	17.8	0.63 (0.51 to 0.79)	74.7	1.49 (1.24 to 1.79)
60–69	22.5	0.78 (0.61 to 0.99)	49.2	1.40 (1.18 to 1.67)	73.4	3.01 (2.47 to 3.65)	29.7	0.82 (0.67 to 1.01)	77.6	1.19 (0.96 to 1.48)	55.8	1.16 (0.96 to 1.42)	25.1	0.88 (0.69 to 1.13)	75.2	1.58 (1.30 to 1.93)
70+	17	0.50 (0.36 to 0.71)	44.9	1.06 (0.80 to 1.39)	65.2	2.47 (1.86 to 3.28)	18.9	0.55 (0.39 to 0.78)	82.3	1.69 (1.24 to 2.31)	49.6	1.07 (0.81 to 1.41)	40.2	1.45 (1.08 to 1.95)	70.6	1.06 (1.00 to 1.78)
Total	25.5	45.7	63.3	33.3	76.9	54.3	23.1	71.4
**Women**																
Wealth quintile*																
Most poor	23.8	1	39.2	1	50.1	1	34.5	1	83.9	1	57.6	1	29.2	1	71.3	1
2	29.1	1.22 (1.01 to 1.49)	44.8	1.28 (1.06 to 1.54)	36.4	0.61 (0.48 to 0.79)	29.7	0.71 (0.56 to 0.90)	86.6	1.30 (1.00 to 1.70)	61.7	1.14 (0.92 to 1.42)	34.7	1.35 (1.07 to 1.71)	72.9	1.10 (0.90 to 1.34)
3	31.7	1.36 (1.12 to 1.65)	47.6	1.43 (1.17 to 1.73)	41.8	0.81 (0.66 to 0.97)	31.4	0.76 (0.63 to 0.93)	87.5	1.42 (1.06 to 1.91)	59.7	1.05 (0.86 to 1.28)	30.9	1.15 (0.94 to 1.41)	74.7	1.22 (0.99 to 1.51)
4	38.5	1.77 (1.46 to 2.15)	48.9	1.48 (1.20 to 1.83)	41.4	0.83 (0.70 to 0.99)	36.6	1.02 (0.82 to 1.28)	83.6	1.09 (0.80 to 1.48)	55.9	0.93 (0.76 to 1.15)	27.7	0.97 (0.76 to 1.25)	74.4	1.25 (1.03 to 1.53)
Least poor	51.4	2.83 (2.28 to 3.52)	58.5	2.14 (1.68 to 2.74)	35.4	0.71 (0.58 to 0.88)	27.3	0.72 (0.57 to 0.90)	83.5	1.20 (0.85 to 1.70)	45.6	0.65 (0.51 to 0.83)	26.1	0.85 (0.65 to 1.11)	74.2	1.33 (1.03 to 1.71)
Age group†																
30–39	41.8	1	42.5	1	19.4	1	37.7	1	81.5	1	56.6	1	24.4	1	66.5	1
40–49	36.2	0.87 (0.75 to 1.01)	49.8	1.38 (1.20 to 1.58)	42.8	2.53 (2.16 to 2.97)	36	0.86 (0.73 to 1.01)	84.2	1.04 (0.86 to 1.25)	58.1	1.02 (0.88 to 1.19)	23.9	0.99 (0.84 to 1.17)	74.9	1.36 (1.17 to 1.58)
50–59	31.8	0.79 (0.66 to 0.95)	52.3	1.60 (1.36 to 1.88)	59	4.37 (3.61 to 5.29)	29.4	0.68 (0.56 to 0.82)	87.6	1.35 (1.04 to 1.75)	53.5	0.86 (0.72 to 1.04)	28.7	1.25 (1.04 to 1.50)	77.2	1.57 (1.32 to 1.86)
60–69	23	0.56 (0.45 to 0.70)	48.6	1.41 (1.15 to 1.73)	62.9	4.59 (3.62 to 5.82)	19.2	0.47 (0.36 to 0.60)	89.4	1.54 (1.12 to 2.12)	56.6	1.03 (0.84 to 1.25)	41.2	1.99 (1.59 to 2.50)	79.3	1.82 (1.45 to 2.27)
70+	18	0.41 (0.29 to 0.59)	53.5	1.67 (1.29 to 2.16)	64.4	4.45 (3.24 to 6.12)	11.7	0.32 (0.21 to 0.48)	93	2.40 (1.41 to 4.08)	52.8	0.97 (0.75 to 1.26)	68.3	5.89 (4.25 to 8.16)	86.8	3.15 (2.13 to 4.66)
Total	34.6		47.6		41.1		31.8		85		56.2		29.8		73.5	

All results weighted and adjusted to reflect the clustered, stratified survey design.

*ORs are adjusted for age group, education, literacy, marital status and occupation.

†ORs are adjusted for wealth quintile, education, literacy, marital status and occupation.

Among men and women, there was a clear increasing trend of overweight or obesity and abdominal obesity (waist to hip ratio) with increasing socioeconomic status—the odds being approximately two to three times higher in the highest socioeconomic group compared with the lowest in both men and women. An apparent decreasing trend in tobacco use, passive smoke exposure and salt consumption was observed with increasing socioeconomic status in men and women, although prevalence remained high in all groups. Fruit and vegetable consumption and physical activity were low and were more evenly distributed across all socioeconomic groups in both men and women. However, men in the least poor group were approximately one-third less likely to be physically inactive compared with men in the most poor group. Clustering of risk factors was high in both sexes, with more than 70% of men and women reporting at least three risk factors. While there was no evidence of the prevalence of risk factor clustering differing by socioeconomic status among men, an apparent increasing trend with increasing wealth appeared among women.

Table 3 and figure 1 also show the prevalence of NCD risk factors and risk factor clustering by age group and sex. Among men and women we see a decreasing trend in overweight and obesity with increasing age, although there is evidence of increasing abdominal obesity in both sexes with increasing age. Current tobacco use was strongly associated with age group, with the odds of tobacco use being 2.5 times and 4.5 times higher among older (70+ years) men and women, respectively, compared with men and women aged 30–39. Conversely, although one-third of men and women were exposed to passive tobacco smoke, the odds of exposure decreased with increasing age.

High prevalence of inadequate fruit and vegetable intake (less than five portions per day) persists across all ages and appears to increase with age group, particularly among women. Similarly, salt consumption was high at all ages. The pattern of low levels of physical activity by age group varied considerably between men and women. For men, three-quarters of men aged 30–39 years engaged in at least 150 min of physical activity per week, and this increased to more than 80% by age 50–60 before declining in older age groups. A similar proportion of younger women engaged in adequate physical activity as their male counterparts, but this decreased sooner and to a greater extent among women as they got older, with only around 30% of women aged 70 years or more maintaining adequate levels of physical activity. Consequently, there was a high prevalence of clustering of risk factors among men and women which increased with age, but women reached and maintained a high prevalence, with approximately three in four women being exposed to at least three risk factors by the age of 40–49 years—higher than men in any age group.

### IFG, IGT and diabetes

**Figure 2:**
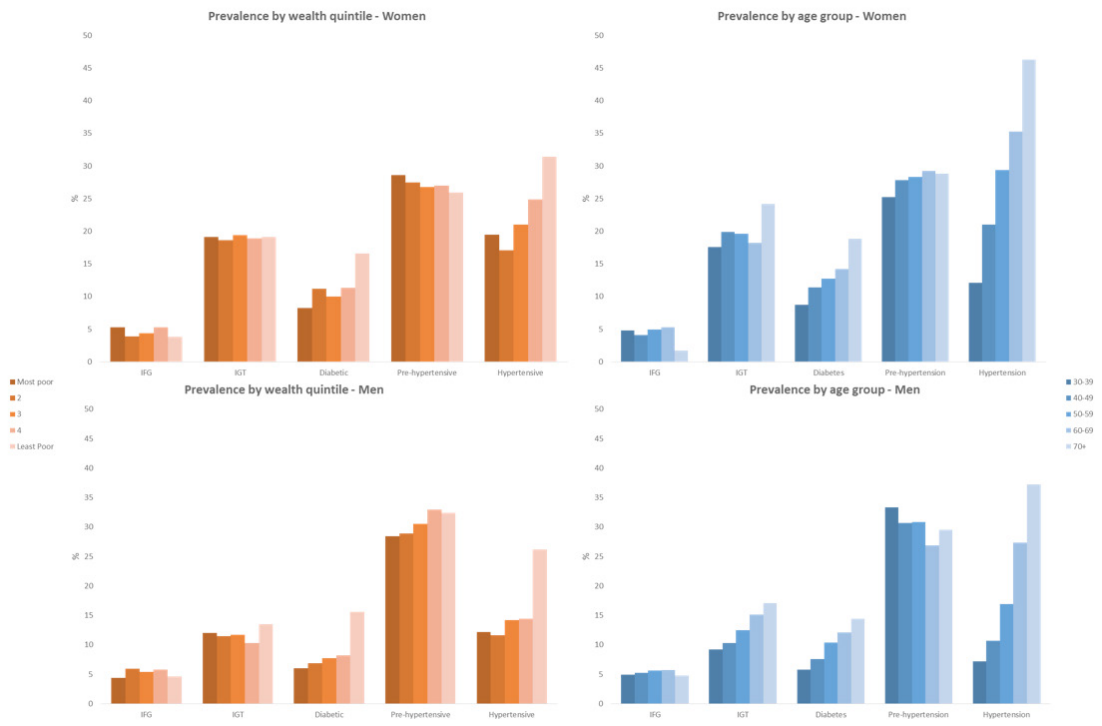
Impaired fasting glucose (IFG), impaired glucose tolerance (IGT), diabetes, prehypertension and hypertension prevalence by wealth quintile, age group and sex among adults aged 30 years and older in rural Faridpur.

The overall prevalence of intermediate hyperglycaemia and diabetes was 17.2% and 8.9% among men and 23.4% and 11.5% among women, respectively (table 4; figure 2). There is no clear evidence of an association between IFG and IGT with wealth quintile among men or women. However the odds of diabetes was approximately 50% higher in men in the least poor group compared with men in the poorest group. The relationship between diabetes and wealth appears to be stronger among women, with those in the lowest wealth quintile having significantly lower odds than most other wealth groups.

**Table 4:** Prevalence and logistic regression ORs of hyperglycaemia and raised blood pressure by sex, wealth quintile and age group among adults aged 30 years and older in rural Faridpur

	Impaired fasting glucose		Impaired glucose tolerance		Diabetic		Prehypertensive		Hypertension	
	%	OR (95% CI)	%	OR (95% CI)	%	OR (95% CI)	%	OR (95% CI)	%	OR (95% CI)
**Men**										
Wealth quintile*										
Most poor	4.4	1	12.0	1	6.0	1	28.4	1	12.2	1
2	5.9	0.94 (0.73 to 1.21)	11.5	0.93 (0.68 to 1.27)	6.9	1.07 (0.75 to 1.52)	28.9	1.02 (0.85 to 1.23)	11.6	0.86 (0.64 to 1.16)
3	5.4	0.98 (0.77 to 1.24)	11.7	0.94 (0.70 to 1.26)	7.7	1.06 (0.75 to 1.51)	30.5	1.10 (0.91 to 1.34)	14.2	0.97 (0.73 to 1.30)
4	5.8	1.07 (0.84 to 1.36)	10.3	0.79 (0.59 to 1.06)	8.2	1.03 (0.73 to 1.48)	32.9	1.22 (1.00 to 1.49)	14.4	0.91 (0.67 to 1.23)
Least poor	4.6	0.84 (0.64 to 1.09)	13.5	0.96 (0.69 to 1.33)	15.6	1.51 (1.06 to 2.16)	32.4	1.19 (0.96 to 1.47)	26.2	1.28 (0.96 to 1.71)
Age group†										
30–39	4.9	1	9.2	1	5.8	1	33.3	1	7.2	1
40–49	5.2	0.80 (0.66 to 0.98)	10.3	1.14 (0.87 to 1.50)	7.6	1.40 (1.03 to 1.91)	30.7	0.89 (0.77 to 1.03)	10.7	1.68 (1.31 to 2.15)
50–59	5.6	0.59 (0.49 to 0.71)	12.5	1.41 (1.10 to 1.81)	10.4	2.07 (1.54 to 2.79)	30.8	0.90 (0.76 to 1.07)	16.9	3.03 (2.30 to 3.98)
60–69	5.7	0.51 (0.41 to 0.63)	15.1	1.63 (1.20 to 2.22)	12.1	2.26 (1.60 to 3.20)	26.9	0.78 (0.64 to 0.95)	27.3	5.09 (3.83 to 6.76)
70+	4.8	0.49 (0.37 to 0.64)	17.1	1.69 (1.15 to 2.49)	14.4	2.32 (1.47 to 3.66)	29.5	0.95 (0.71 to 1.26)	37.2	6.22 (4.36 to 8.86)
Total	5.2		11.8		8.9		30.7		15.8	
**Women**										
Wealth quintile*										
Most poor	5.3	1	19.1	1	8.2	1	28.6	1.00	19.5	1.00
2	3.9	0.71 (0.47 to 1.07)	18.6	0.98 (0.79 to 1.21)	11.2	1.41 (1.04 to 1.89)	27.5	0.97 (0.81 to 1.18)	17.1	0.88 (0.70 to 1.12)
3	4.4	0.81 (0.53 to 1.24)	19.4	1.03 (0.83 to 1.27)	10	1.23 (0.92 to 1.65)	26.8	0.94 (0.79 to 1.13)	21	1.16 (0.93 to 1.45)
4	5.3	1.01 (0.66 to 1.54)	18.9	0.99 (0.79 to 1.25)	11.3	1.43 (1.10 to 1.87)	27.0	0.95 (0.80 to 1.14)	24.9	1.43 (1.17 to 1.76)
Least poor	3.8	0.70 (0.45 to 1.09)	19.1	1.02 (0.81 to 1.29)	16.6	2.22 (1.64 to 3.00)	25.9	0.92 (0.75 to 1.13)	31.4	1.87 (1.51 to 2.32)
Age group†										
30–39	4.8	1	17.6	1	8.7	1	25.2	1.00	12.1	1.00
40–49	4.1	0.84 (0.61 to 1.16)	19.9	1.15 (0.97 to 1.36)	11.4	1.37 (1.07 to 1.75)	27.8	1.12 (0.95 to 1.32)	21.0	2.08 (1.71 to 2.53)
50–59	4.9	1.01 (0.68 to 1.51)	19.6	1.08 (0.86 to 1.36)	12.7	1.63 (1.24 to 2.15)	28.3	1.12 (0.93 to 1.35)	29.4	3.45 (2.79 to 4.26)
60–69	5.3	1.08 (0.69 to 1.69)	18.2	0.92 (0.73 to 1.16)	14.2	2.02 (1.45 to 2.81)	29.2	1.17 (0.91 to 1.50)	35.3	4.67 (3.64 to 5.98)
70+	1.7	0.32 (0.13 to 0.78)	24.2	1.23 (0.90 to 1.67)	18.8	2.88 (1.89 to 4.38)	28.8	1.15 (0.84 to 1.58)	46.3	7.04 (5.11 to 9.69)
Total	4.5		19.0		11.4		27.2		22.6	

All results weighted and adjusted to reflect the clustered, stratified survey design. Note missing blood glucose data for three cases.

*ORs are adjusted for age group, education, literacy, marital status and occupation.

†ORs are adjusted for wealth quintile, education, literacy, marital status and occupation.

With regard to age group, there is evidence of increasing hyperglycaemia with increasing age among men such that the prevalence rose from approximately one in five men aged 30–39 years to more than one in three men aged 60 years and above. A similar trend is observed for women, although at a higher level, with 45% of women aged 70 years or above having IFG, IGT or diabetes (table 4; figure 2).

### Raised blood pressure

Almost half of men and women aged 30 years and above in our study sample were categorised as prehypertensive or hypertensive (table 4; figure 2). There is no strong evidence of an association between these hypertensive measures and wealth quintile among men. There is no evidence of an association between wealth quintile and prehypertension among women, but an increasing trend in hypertension with increasing wealth is observed, with women in the two least poor quintiles being 43% and 87% more likely to have hypertension compared with women in the poorest group, respectively.

Age is much more clearly associated with hypertension among men and women, although there is little evidence of effect of age on prehypertension (table 4; figure 2). Although starting from different baseline prevalences in the youngest age groups, the prevalence of hypertension rose higher and at younger ages among women than men such that there was approximately a doubling of odds of hypertension among women between 30–39 years and 40–49 years, with significant but smaller incremental increases for every subsequent age group.

### Diabetes and hypertension status awareness, treatment and control

**Figure 3:**
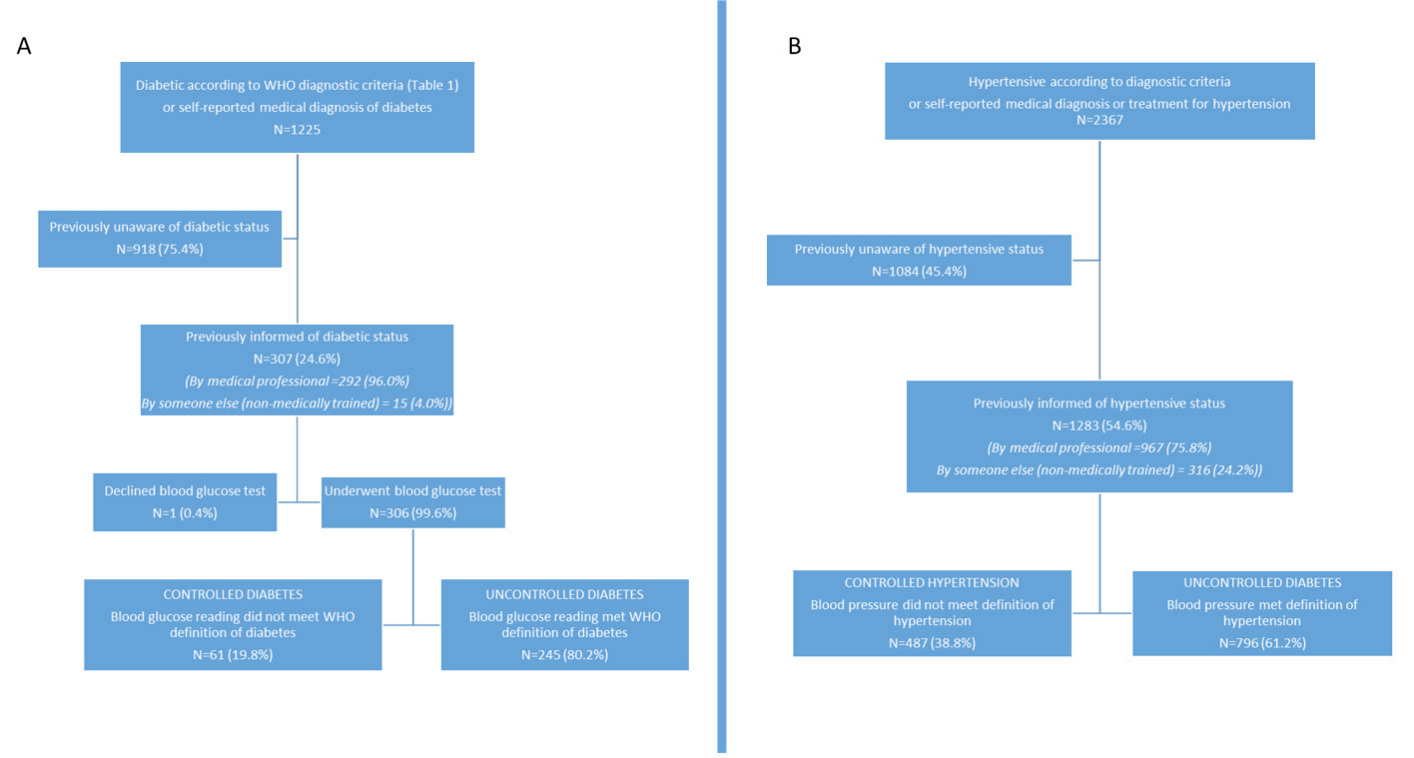
Diabetes (A) and hypertension (B) awareness and control among our study population identified with these conditions in rural Faridpur.

Among the 1225 individuals identified to be diabetic, only 307 individuals (24.6%) were already aware of their diabetic status. Of these 307, 95% (n=292) had been informed of their status by a trained medical professional. Women with diabetes were 37% less likely than men to know that they were diabetic (OR 0.63 (95% CI 0.48 to 0.81)). Of those who had been informed by a trained medical professional prior to our survey, 252 (86.1%) reported ever receiving medical treatment or advice. There was no evidence of a difference between women and men in the odds of receiving treatment (OR 0.79 (95% CI 0.41 to 1.55). Of the 307 individuals with diabetes who reported prior awareness of their diabetic status, 306 underwent blood glucose testing in our survey and 245 (80.2%) of these had fasting plasma glucose ≥7 mmol/L or 2-hour post glucose load plasma glucose ≥11.1 mmol/L, indicating suboptimal control of the condition (figure 3A).

Of the 2367 individuals identified with hypertension through our measurements, 1283 (54.6%) reported that they aware that they had hypertension prior to our survey, with 967 (75.8%) of these individuals having previously been informed by a trained medical professional. In contrast to diabetes, women with hypertension were more likely to be aware of their status than men (OR 1.74 (95% CI 1.44 to 2.09)). Of those who had been informed of their hypertensive status by a trained medical professional prior to ours, 87.3% reported ever receiving medical treatment or advice, with no apparent differences between women and men, 1.10 (95% CI 0.74 to 1.64). Of the 1283 individuals with hypertension who reported prior awareness of their hypertensive state, 61.2% (n=796) had raised blood pressure (systolic blood pressure ≥140 mm Hg or diastolic blood pressure ≥90 mm Hg) at the time of our survey (figure 3B).

## Discussion

Between a quarter and one-third of adults aged 30 years or older in rural Bangladesh have IFG, IGT or diabetes, and approximately half of all adults have raised blood pressure that meets the standard definition of prehypertension or hypertension. Despite these high burdens of raised blood sugar and blood pressure, three-quarters of individuals with diabetes and almost half of the individuals with hypertension were unaware of their status prior to our survey. Women are more affected than men in terms of raised blood sugar and raised blood pressure, as well as in terms of most NCD risk factors. Nevertheless, a high prevalence of common NCD risk factors persists across age, wealth and sex groups, with almost three-quarters of the adult population aged 30 years or older reporting three or more risk factors. This clustering of factors raises risk by more than a summation of findings,^7^ so the observed burden of dysglycaemia and raised blood pressure is likely to increase in the immediate future. Overall, these data describe a huge unmet need for prevention, care-seeking and control of NCDs in rural Bangladesh, with likely consequences on the individuals affected and the wider household, community and economic environment in which these individuals live.^37^

Our large, population-based sample size, randomly selected from a contemporary and purpose-made sampling frame, rigorous fieldworker training, and data quality control measures are major strengths of our study. Direct measurement of fasting and 2-hour post glucose load blood sugar levels using capillary whole blood was logistically more complicated than random blood glucose or fasting-only measures as is commonly used in community-based surveys in low-income settings, but increases confidence in our population estimates of IFG, IGT and diabetes prevalence. Nevertheless, despite having been acceptable for epidemiological studies,^38 39^ capillary blood glucose levels can overestimate blood sugar compared with venous samples, and so our prevalence estimates may be higher than would be achieved using more stringent clinical methods. Prevalence estimates may also have been affected by including individuals with self-reported prior medical diagnosis of diabetes and hypertension in the numerator. The validity of these self-reports or the diagnostic criteria used in earlier diagnoses is unknown.

Given the time commitment and inconvenience asked from respondents, our response rate of almost 90% is exceptionally good. Nevertheless, we did observe some potential response bias in terms of age, with male non-responders being younger and female non-responders being older than their responding counterparts. This likely reflects the fact that younger men were less able to commit the time required for our survey due to work commitments, whereas older women may simply have been less inclined to participate. The consequence of these potential response biases is that our findings may under-represent the younger male population and so potentially overestimate the overall burden of risk factors and disease associated with increasing age. Conversely, our data may under-represent older women and so underestimate the overall burden given that we see increasing prevalence of risk factors and disease with age group.

The cross-sectional design and contemporary measurement of socioeconomic status, age and NCD risk factors must also influence our interpretation of observed associations and limit inferences of cause and effect. Furthermore, although homogeneity in rural Bangladeshi populations has been noted by others,^40^ extrapolation of our findings to other rural areas must be done with caution, and our findings are likely to be different from those from an urban or mixed population. Finally, our large sample size, based on trial assumptions of effect size, prevalence and intracluster correlation, may increase the risk of type 1 one errors. As such, we have taken care to present and interpret measures of associations in terms of their magnitude and trends, with emphasis on public health significance rather than statistical significance.

High BMI is an important risk factor for NCDs, and halting its increasing prevalence is included in the WHO Global Action Plan for the prevention and control of NCDs.^41^ Overweight and obesity have been shown to increase the risk of NCDs, and abdominal obesity in particular is associated with increased risk of metabolic syndrome and diabetes.^42–45^ The high burdens of overweight and obesity among younger age groups are alarming and, given the majority of risk factors increase with age, set an unhealthy basis on which further risk may be added. Being underweight is also associated with increased risk of morbidity and mortality, a so-called J-shaped association.^46^ In our data we found that 25% of underweight individuals (BMI<18.5) had intermediate hyperglycaemia or diabetes, and around 38% had raised blood pressure (data not shown). Public health and policy approaches to NCD prevention must therefore recognise this complex relationship between risk factors and disease, rising to the challenge of tailoring dietary advice appropriately for heterogeneous at-risk populations, especially in settings where household food insecurity remains an issue.^47^

The WHO recommends intake of a minimum of 400 g of fruits and vegetables per day, citing evidence that this can help to reduce the risk of obesity, diabetes and cardiovascular diseases.^48^ To promote this behaviour, 400 g of fruit and vegetables has frequently been translated into five portions of fruits or vegetables per day. Based on this cut-off, our data show very high levels of inadequate fruit and vegetable consumption across all ages and wealth groups and men and women, although there is an apparent trend in increasing inadequate intake with age. It is known that an individual’s likelihood of achieving ‘five-a-day’ is affected by many individual-level and household-level factors, one of the most important of which is socioeconomic status.^49^ Indeed, our analysis by wealth quintile does indicate an increasing trend in fruit and vegetable consumption with increasing wealth, although this is not statistically significant and, even in the least poor group, only around 27% of men and 16% of women achieve the recommended daily intake. In interpreting the data on fruit and vegetable consumption, however, it is important to note that respondents generally found the WHO STEPS question on consumption on a ‘typical day’ in a ‘typical week’ difficult to conceptualise, reflecting temporal variations in fruit and vegetable availability and affordability within this population.

High dietary salt intake is an important risk factor for NCDs, especially hypertension. It has been identified as an indicator of the NCD Global Monitoring Framework, which targets a 30% relative reduction of dietary salt between 2010 and 2025. Progress towards this target requires quantification of salt intake. Few studies have attempted to do this in Bangladesh, and the quality of estimates is limited by selection bias, small sample sizes or reliance on production and sales data. Nevertheless, the available data suggest an average salt intake of between 15 g/day and 21 g/day,^50–52^ three to four times higher than the recommended 5 g daily limit.^48^ We did not attempt to quantify salt intake in our study but did ask respondents whether they added salt to their food after it was cooked and how often, with approximately 50% of all age and wealth groups reporting that they add salt to every meal. The addition of salt to food ‘at the table’ is considered a primary target for salt reduction,^50^ and campaigns are under way in Bangladesh. The activities undertaken by the National Heart Foundation of Bangladesh for salt reduction include raising awareness through organising seminars in different areas of the country, holding consultative meetings with stakeholders including the food and beverage industry, as well as generating evidence through surveys and studies, for example.^53^ Policy support that stresses the importance of salt reduction and addresses salt content of processed foods by formulating appropriate laws and regulations, setting goals, and supporting monitoring and evaluation is lacking.^53^

There is good evidence that regular physical activity of at least 150 min of moderate-intensity to vigorous-intensity per week reduces rates of all-cause mortality and a number of NCDs.^54^ Even relatively small amounts of physical activity such as walking are shown to provide health benefits among high-risk adults, including those with hypertension, diabetes and/or high BMI.^55^ Our data show that between one-fifth and a third of men and women do not achieve these levels of physical activity. These estimates are slightly lower than the estimates of 35%–38% physical inactivity among Bangladeshi adults aged 25 years and older based on the 2010 WHO STEPS survey.^7 56^ These differences may possibly be explained by the exclusively rural population of our study compared with the mixed (rural and urban) STEPS survey. Women are at greater risk of physical inactivity overall and become increasingly inactive at younger ages than men. This likely reflects the distinct gender roles and cultural norms of physical activity within this rural context. Among women, the levels of physical activity decrease significantly from age 50 years and above, while no significant difference is observed among men until age 70 and above. Evidence of association with wealth group is not particularly strong, although it does appear that physical activity levels are better among the least poor men and women relative to the poorest group.

Our data on tobacco use support previous estimates in Bangladesh^57^ and reveal exceptionally high use among men and women, although mode of use differs between sexes, with more women chewing tobacco or using snuff than men (figure 2). Correspondingly, passive exposure to tobacco smoke is high, although it decreases with age—this reflects cultural norms of not smoking in front of one’s elder. A decreasing trend in tobacco use with wealth is apparent, the reasons for which are unclear. The decreasing trend with wealth, also observed with passive smoke exposure, salt consumption and clustering of risk factors, may reflect greater awareness, motivation and opportunity to avoid risk in wealthier individuals, although further research is required to explore this. In 2013, the National Assembly of Bangladesh passed the Tobacco Control Law Amendment Bill, which was considered a major step forward in tobacco control measures as it included smokeless tobacco products and the expansion of public places that are to be smoke-free. Further community-based behaviour change and smoking cessation initiatives are required to complement such governmental initiatives.

Our estimates of intermediate hyperglycaemia, diabetes and raised blood pressure add to the range of estimates for Bangladesh, being comparable, although slightly higher than the BDHS diabetes estimates^9^ and the Bangladesh NCD Risk Factor Survey estimates of hypertension.^7 21–27^ Our higher estimates may be an artefact of methodological differences or may represent the increasing burden of disease in this context. Within the context of high prevalence of disease, awareness and control of one’s own diabetic and hypertensive state remain low and are comparable with that reported for hypertension elsewhere in Bangladesh and other South Asian populations.^58^ It is not surprising that the 2010 Bangladesh NCD Risk Factor Survey found only 4% of adults aged 25 years or older with a self-reported diagnosis of diabetes by a health professional.^8^ Differences observed in awareness of hypertension between the sexes may reflect greater rates of detection among women during antenatal care or other care-seeking during pregnancy and childbirth which, depending on the availability and quality of services, may also offer opportunities for greater detection of diabetes among women. Increasing detection among men and women however remains a challenge that requires interventions that raise overall awareness of the risk factors, signs and symptoms of both conditions, inform populations about opportunities and benefits of monitoring one’s own health, and address limited opportunities for testing within public-sector and private-sector health providers located in rural areas.

## Conclusion

NCD risk factors, hyperglycaemia and raised blood pressure are an immediate and often unrecognised health threat in rural Bangladesh. There is an urgent need for gender-sensitive health service and community-based initiatives to improve detection and treatment strategies while also preventing the growing disease burden.
